# A cluster-randomized controlled trial of a nurse-led artificial intelligence assisted prevention and management for delirium (*AI-AntiDelirium*) on delirium in intensive care unit: Study protocol

**DOI:** 10.1371/journal.pone.0298793

**Published:** 2024-02-29

**Authors:** Shan Zhang, Wei Cui, Shu Ding, Xiangyu Li, Xi-Wei Zhang, Ying Wu

**Affiliations:** 1 School of Nursing, Capital Medical University, Beijing, China; 2 Cardiology Department, Beijing Chao-Yang Hospital, Capital Medical University, Beijing, China; 3 Nursing Department, Anzhen Hospital, Capital Medical University, Beijing, China; Centre Hospitalier Universitaire Farhat Hached de Sousse, TUNISIA

## Abstract

**Background:**

Delirium is a common complication among intensive care unit (ICU) patients that is linked to negative clinical outcomes. However, adherence to the Clinical Practice Guidelines for the Prevention and Management of Pain, Agitation/Sedation, Delirium, Immobility, and Sleep Disruption in Adult Patients in the ICU (PADIS guidelines), which recommend the use of the ABCDEF bundle, is sub-optimal in routine clinical care. To address this issue, *AI-AntiDelirium*, a nurse-led artificial intelligence-assisted prevention and management tool for delirium, was developed by our research team. Our pilot study yielded positive findings regarding the use of *AI-AntiDelirium* in preventing patient ICU delirium and improving activities of daily living and increasing intervention adherence by health care staff.

**Methods:**

The proposed large-scale pragmatic, open-label, parallel-group, cluster randomized controlled study will assess the impact of *AI-AntiDelirium* on the incidence of ICU delirium and delirium-related outcomes. Six ICUs in two tertiary hospitals in China will be randomized in a 1:1 ratio to an *AI-AntiDelirium* or a PADIS guidelines group. A target sample size of 1,452 ICU patients aged 50 years and older treated in the ICU for at least 24 hours will be included. The primary outcome evaluated will be the incidence of ICU delirium and the secondary outcomes will be the duration of ICU delirium, length of ICU and hospital stay, ICU and in-hospital mortality rates, patient cognitive function, patient activities of daily living, and ICU nurse adherence to the ABCDEF bundle.

**Discussion:**

If this large-scale trial provides evidence of the effectiveness of *AI-AntiDelirium*, an artificial intelligence-assisted system tool, in decreasing the incidence of ICU delirium, length of ICU and hospital stay, ICU and in-hospital mortality rates, patient cognitive function, and patient activities of daily living while increasing ICU nurse adherence to the ABCDEF bundle, it will have a profound impact on the management of ICU delirium in both research and clinical practice.

**Clinical trial registration:**

ChiCTR1900023711 (Chinese Clinical Trial Registry).

## Introduction

Delirium is a preventable neuro-psychiatric syndrome [1], and common complication in critically ill patients in intensive care units (ICUs), affecting 70%–87% of all patients in ICUs [2, 3]. ICU delirium can result in increased length of hospital stay, mechanical ventilation (MV) days, and mortality [2, 4], negative outcomes that are independently associated with the duration of ICU delirium [4, 5]. Therefore, interventions are critical to prevent ICU delirium and eliminate its adverse consequences [6].

Although the mechanism underlying ICU delirium is not yet fully understood, a combination of multiple risk factors has been proposed to contribute to its incidence [7, 8]. To address these risk factors, the Clinical Practice Guidelines for the Prevention and Management of Pain, Agitation/Sedation, Delirium, Immobility, and Sleep Disruption in Adult Patients in the ICU (PADIS guidelines) were developed by the Society of Critical Care Medicine [6]. These guidelines recommend the use of the ABCDEF bundle approach consisting of (A) assess, prevent, and manage pain; (B) both spontaneous awakening trials and spontaneous breathing trials; (C) choice of analgesia and sedation; (D) delirium assessment, prevention, and management; (E) early mobility and exercise; and (F) family engagement and empowerment. This bundle aims at minimizing modifiable risk factors to decrease the onset of ICU delirium.

Several well-designed randomized clinical trials (RCTs) have validated the effectiveness of bundle interventions in eliminating risk factors to prevent the onset of delirium or shortening its duration [9–11]. However, a meta-analysis of the effectiveness of bundle interventions in reducing the incidence and decreasing the duration of ICU delirium by our team failed to support the aforementioned outcomes [12]. Several factors may explain these mixed findings. First, although the use of all elements of the ABCDEF bundle are suggested by the PADIS guidelines to eliminate modifiable risk factors for ICU delirium [6], many studies only used selected elements [13, 14]. Second, adherence to the bundle among nursing staff is poor due to the complexity of the bundle components [15, 16]. Previous studies have demonstrated that diverse barriers may impede adherence to the bundle, such as heavy workload, the need to maintain detailed records [17], the complex algorithm used in the assessment tools [18, 19], and the difficulty in collecting data on and monitoring numerous risk factors through multiple channels [20]. Hence, it is important to develop a tool to simplify and reduce the workload to enhance adherence among nursing staff.

Reflecting the rapid development of information technology, the Clinical Decision Support System (CDSS) was developed. Widely used in most hospitals worldwide for disease assessment, management, and record keeping [21, 22], the CDSS can collect, sort, classify, and establish a logical relationship among patient data. It also allows the use of health alerts and provides feedback for decision support during disease diagnosis, treatment, and nursing activities [23, 24]. By such means, the use of CDSS can help medical staff reduce their workload and improve adherence in implementing nursing interventions [25, 26].

To reduce the incidence of ICU delirium and improve adherence to the ABCDEF bundle among ICU nurses, our research team developed the Artificial Intelligence Assisted Prevention and Management for Delirium (*AI-AntiDelirium*) platform based on the PADIS guidelines and the principles of system design [6, 27]. The development process of the *AI-AntiDelirium* tool using the Template for Intervention Description and Replication (TIDieR) checklist was described in our previous work [28], in which we described needs assessment, development of interventions, and formulation of the *AI-AntiDelirium* database.

The *AI-AntiDelirium* tool consists of four functional modules: the *delirium assessment module*, the *delirium risk factors assessment module*, the *nursing care plan module*, and the *care activity checklist module*. For the delirium assessment module, our research team developed the intelligent Confusion Assessment Method for the Intensive Care Unit (iCAM-ICU), a CDSS-assisted delirium screening tool found to have high sensitivity and specificity for use by bedside nurses, as validated in a study published elsewhere [29]. The Chinese version of the CAM-ICU was validated in a study of Chinese ICU patients that found it had a sensitivity and specificity of 91.8% and 90.8%, respectively, indicating that it has high accuracy in detecting ICU delirium when used by nurses in their routine practice [30].

The second module, the delirium risk factors assessment module, calculates the possibility of delirium according to the prediction model by automatically obtaining risk factor information [31]. The third module, the nursing care plan module, provides a personalized ICU delirium care plan based on the risk factors identified. The fourth module, the care activity checklist module, allows the development of an individualized care activity checklist with intervention duration, intensity, or dose based on the personalized care plan.

Our pilot study indicated that the use of the *AI-AntiDelirium* tool reduces the incidence of ICU delirium. This cluster randomized controlled trial (CRCT) aims to determine the effects of the use of *AI-AntiDelirium* in decreasing the incidence of ICU delirium and several secondary outcomes, including the duration of ICU delirium, length of ICU and hospital stay, mortality, and other delirium-related outcomes. We hypothesize that the use of *AI-AntiDelirium* will decrease the incidence of ICU delirium, duration of ICU delirium, length of ICU and hospital stay, and ICU and in-hospital mortality rates while improving cognitive function and activities of daily living compared with the use of the PADIS guidelines. If this large-scale trial demonstrates its effectiveness in reducing the incidence of ICU delirium and promoting adherence to implementation, *AI-AntiDelirium* will have a profound impact on the management of ICU delirium in both research and clinical practice.

## Materials and methods

This study will be conducted following the Standard Protocol Items of the Recommendations for Interventional Trials (SPIRIT; S1 File) [32].

### Study design

**Fig 1:**
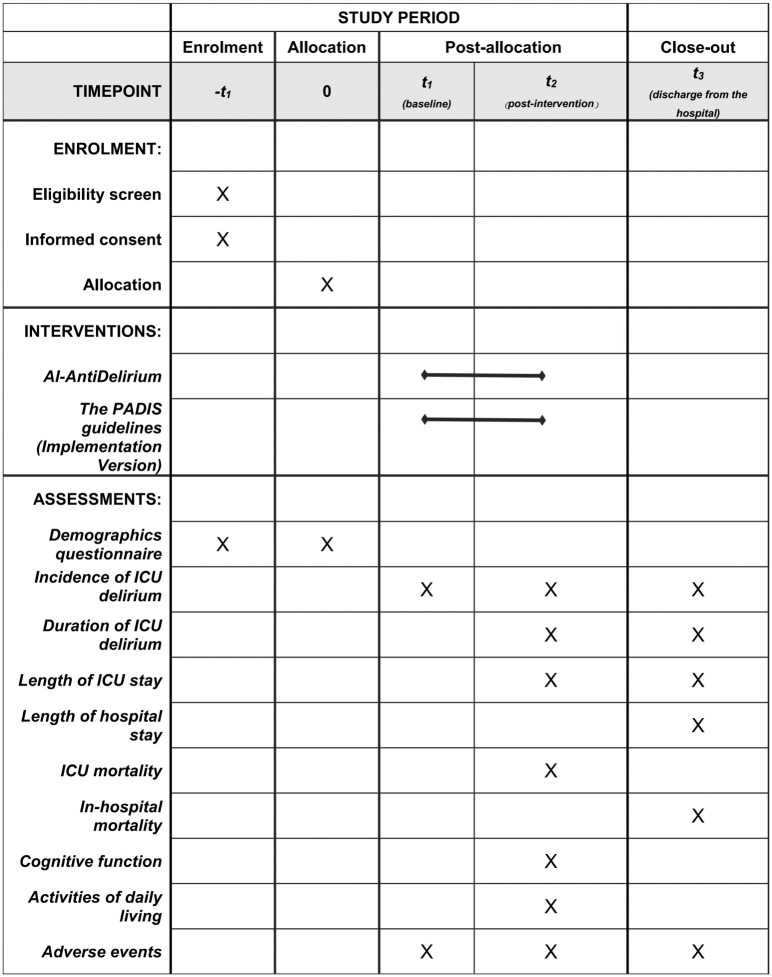
Schedule of enrolment, interventions, and assessments. *AI-AntiDelirium*: the **A**rtificial **I**ntelligence **A**ssisted Preve**nti**on and Management for **Delirium**, *PADIS guideline*: the Pain, Agitation, Delirium, Immobility, and Sleep (PADIS) Guidelines.

This study will be a large-scale, pragmatic, open-label, cluster RCT in which six ICUs will be randomized in a 1:1 ratio to the *AI-AntiDelirium* group or to the PADIS guidelines group (Fig 1).

### Study setting

**Fig 2:**
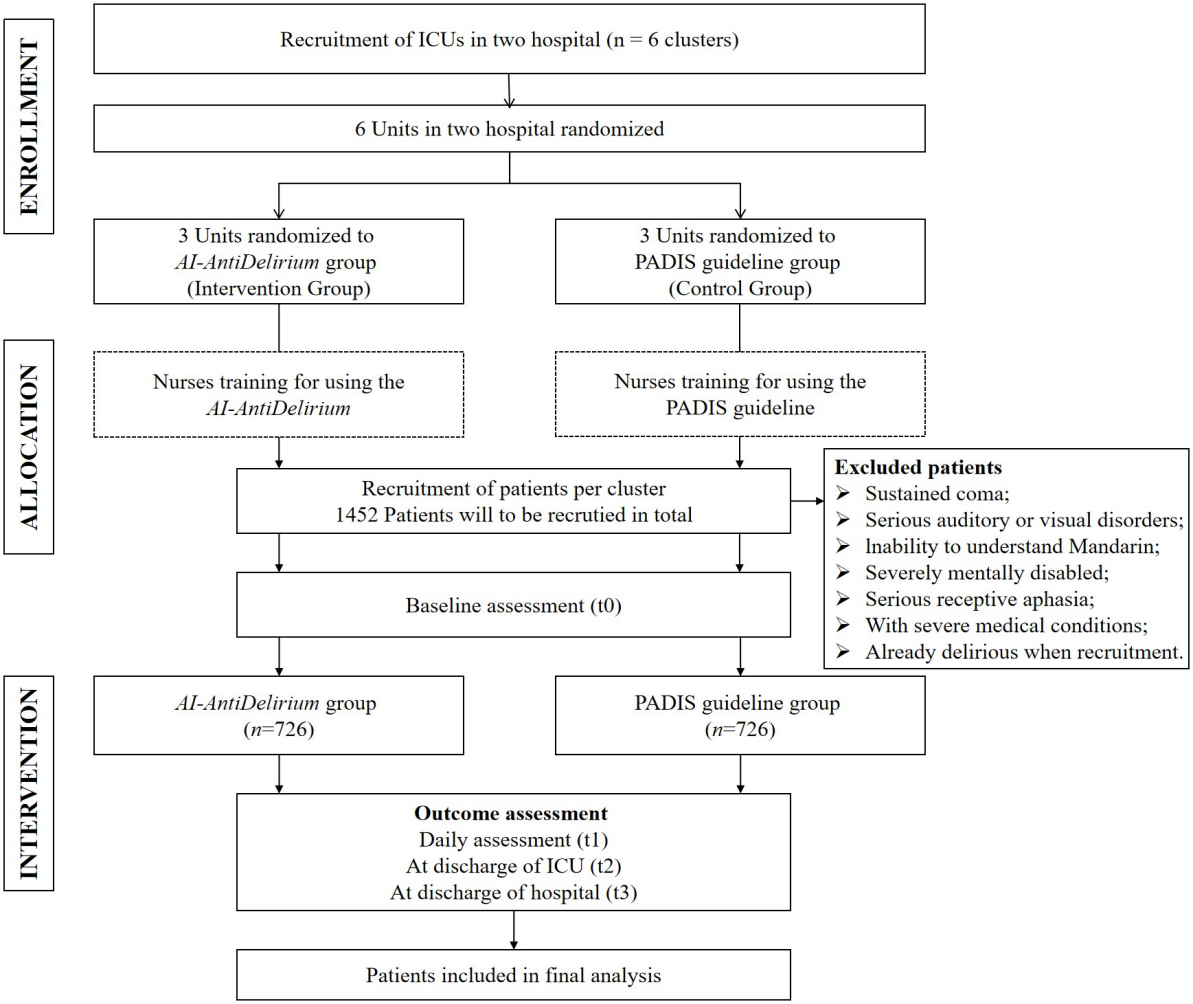
Flowchart of the study.

The study will be conducted in six ICUs in two tertiary hospitals (Fig 2). Each ICU has over 10 beds and typically admits approximately 100 patients per month, providing a sufficient sample size for the study. Based on preliminary data obtained from similar ICUs [31], a 30% incidence reduction in the intervention group is expected.

### Participants

A consecutive sample of patients will be screened and recruited. The inclusion criteria will be (1) age 50 years or older and (2) expected ICU stay of at least 24 hours. The exclusion criteria are (1) non-emergence from coma (Richmond Agitation Sedation Scale [RASS] score = –4 to –5) during ICU stay; (2) unable to understand Mandarin; (3) severe mental disabilities, Wernicke’s aphasia, or other medical conditions, such as active bleeding, spinal cord injury, and open lumbar drainage, that prevent initiation of physical therapy or contraindicate of therapy; (4) participation in other ongoing trials; and/or (5) presence of delirium at baseline evaluation.

Our target sample size is 205 patients from each ICU to provide a power of 80% and a two-sided significance level (α) of 0.05 with an intra-cluster (within-unit) correlation of 0.001 [33, 34]. Considering a likely dropout rate of 15% based on previous studies, enrolment of 242 patients per cluster for a total of 1,452 (242 × 6) patients is expected.

### Recruitment

All recruitment will be conducted by trained researchers who will not be involved in the intervention and are blinded to the patient groups. To identify eligible patients, researchers will screen patients based on the inclusion and exclusion criteria. All eligible patients or their immediate relatives will sign an informed consent.

### Randomization and blinding

After agreeing to participate in the current study, the eligible ICUs (clusters) will be randomized based on their size, types of diseases treated, and incidence of delirium among their patients. ICU patients in two tertiary care hospitals will be randomized 1:1 to receive either *AI-AntiDelirium* or PADIS guidelines care. The allocation sequence will be performed by a research staff member independent of data collection or analyses. The statisticians and ICU staff (nurse administrators and bedside nurses who will deliver the intervention) will be masked to the study hypothesis and specific protocols of the *AI-AntiDelirium* tool. By the nature of the interventions, it will not be feasible to blind ICU patients and study researchers. To achieve as much objectivity as possible, the study researchers will use standardized tools to collect data.

### Intervention group

Patients in the intervention group will receive *AI-AntiDelirium* care daily after baseline assessment until ICU discharge or death. The intervention will be mainly implemented by bedside nurses, who will be trained in the use of *AI-AntiDelirium* to prevent delirium for 2 weeks before the intervention begins. The staff will install the *AI-AntiDelirium* platform on their personal digital assistant (PDA) and be provided with multi-modal education to familiarize them with all procedures. The specific intervention processes will be *ICU delirium assessment*, *risk factor assessment*, and *tailored interventions*. For ICU delirium assessment, bedside nurses will use the iCAM-ICU module of *AI-AntiDelirium* to assess ICU delirium at least two times daily, once between 8 a.m. and 10 a.m. and once between 4 p.m. and 6 p.m. After the assessment has been completed, the system will automatically indicate whether the patient has delirium.

For risk factor assessment, bedside nurses will apply the risk factor assessment module to assess whether patients have one or more of the following 10 delirium-related risk factors: (1) hearing impairment, (2) vision deficits, (3) pain, (4) use of sedatives or analgesics, (5) use of mechanical ventilation, (6) use of indwelling catheter, (7) infection, (8) immobility, (9) sleep disturbance, and (10) lack of family companionship. Risk levels among ICU patients will be predicted using the dynamic delirium prediction rule in patients admitted to ICUs (DYNAMIC-ICU) [31].

For development of tailored interventions, the nursing care plan module will automatically generate individualized ICU delirium measures according to existing risk factors (Table 1). ICU nurses will review all interventions for each patient to determine whether the nursing care plan is feasible. The care activity checklist module will automatically provide the schedule for each intervention and its duration, intensity or dose, and provider based on the individualized nursing care plan. The nurses will implement individual interventions during their entire shift. After the nurses click on the intervention button on the PDA after completing the intervention, the *AI-AntiDelirium* tool automatically records their names and the time of completion.

**Table 1:** Interventions targeting risk factors.

Risk Factor	Intervention
Hearing impairment	Speak loudly, slowly, and patiently Assist patients in wearing hearing aids correctly
Visual impairment	Remind family members to bring eyeglasses to the ICU at next visit Assist patients in wearing eyeglasses correctly
Pain	Provide non-pharmacological interventions, such as distraction and relaxation therapy Use analgesics as prescribed
Use of anesthesia or sedatives	Adjust sedative dose according to RASS and maintain light sedation (RASS score ≥ –2) Implement spontaneous awakening trial
Mechanical ventilation	Conduct spontaneous breathing trials as prescribed Monitor patient’s respiratory status
Indwelling catheter	Remove catheter as soon as possible Conduct timed urination
Infection	Reduce invasive surgery Avoid unnecessary catheterization Advise doctors to remove catheter as soon as possible
Immobility	Level 0: Advise patients to rest in bed and restrict activity Level 1: Help patients perform passive range-of-motion exercises, 10 times for each joint Level 2: Help patients perform active range-of-motion exercises in bed for 10–20 min Level 3: Help patients sit on bedside for 20 min; Level 4: Assist patients to stand still at the bedside for 5–10 min Level 5: Assist patients to walk along the aisle for 5–10 min
Sleep deprivation	Reduce duration of sleep during the day by 1 or 2 hours Assist patients to wear earplugs or anti-noise equipment Assist patients in wearing eye masks
No visits by family members	Encourage family visits

### Control group

Patients in the control group will receive nursing care based on the PADIS guidelines (S2 File) until ICU discharge or death. The intervention will be implemented by bedside nurses after they have been trained in using the PADIS guidelines based on the ABCDEF bundle, which has the same contents as the *AI-AntiDelirium* tool (S2 File). The specific intervention processes are ICU delirium assessment, risk factor assessment, and development of tailored interventions. For the ICU delirium assessment, nurses will assess ICU delirium using the CAM-ICU at least twice a day between 8 a.m. and 10 a.m. and between 4 p.m. and 6 p.m. and record the results manually. For the risk factor assessment, the bedside nurses will complete the paper-based ICU delirium risk factor assessment checklist at least once a day between 8 a.m. and 10 a.m. The nurses will individually assess patients’ current risk factors and manually calculate their risk of developing ICU delirium. For the development of tailored interventions, the nurses will extract nursing intervention data from the delirium prevention and management interventions of the PADIS guidelines (implementation version) according to the risk factors, record the interventions in nursing care records, and implement an appropriate subset of interventions.

**Fig 3:**
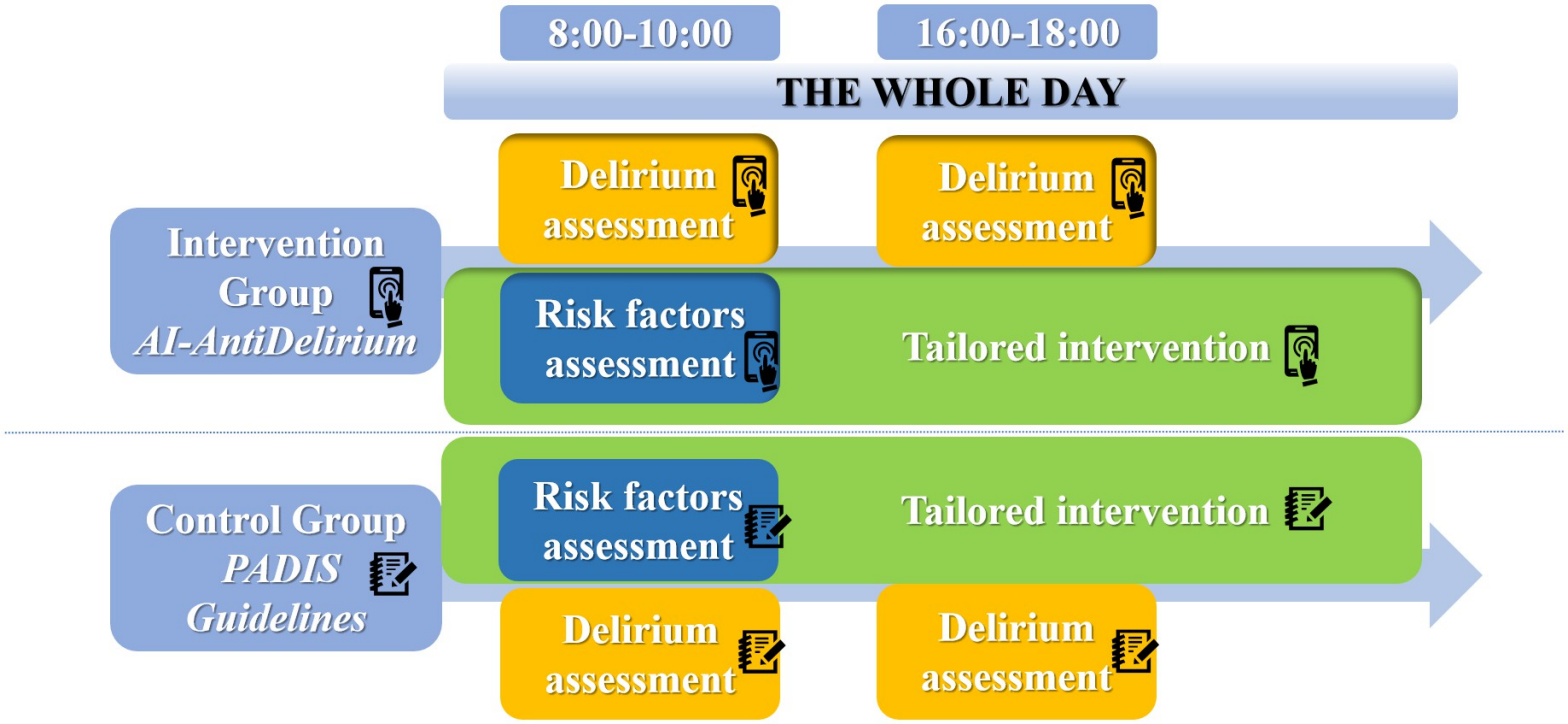
The nurse workflow in the intervention group and control group.

Fig 3 shows the workflow the bedside nurses will follow in using *AI-AntiDelirium* (intervention group) or the PADIS guidelines (control group) to prevent ICU delirium. The intervention will be integrated into daily patient care activities and delivered by bedside nurses at various times each day. Safety will be monitored continuously throughout the study.

### Outcome measures

The primary outcome will be the incidence of ICU delirium, which will be assessed twice daily using the CAM-ICU by two trained outcome assessors (research staff) blinded to patient assignment in each hospital. Delirium will defined as a CAM-ICU score of at least +1 during the day. The secondary outcomes of the study will be the duration of ICU delirium, defined as the number of days a patient has a positive CAM-ICU score while in the ICU; length *of ICU stay*, defined as the number of days from ICU admission to ICU discharge; length *of hospital stay*, defined as the number of days from hospital admission to hospital discharge; *ICU mortality*, defined as the rate of death during ICU stay; and in-*hospital mortality*, defined as the rate of death from any cause during hospital stay. *Cognitive function* will be measured using the Mini-Mental State Examination (MMSE) [35] upon ICU discharge. The MMSE score for each item ranges from 0 (poor) to 30 (excellent), and the total score classifies patents as having definitive impairment (<23), mild impairment (24–27), or no impairment (28–30). The Chinese version of the MMSE has been validated and found to have acceptable validity and reliability [36].

*Activities of* daily living will be measured using the Barthel Index (BI) [37] upon ICU discharge. The BI has 10 items assessing the capacity to perform daily activities, including feeding, bathing, grooming, dressing, controlling stool and urine, using the toilet, transferring from bed to chair, walking, climbing, and descending stairs, to obtain a score ranging from 0 to 100, with lower scores indicating increased dependence. The Chinese version of the BI has shown acceptable validity and reliability in measuring the activities of daily living [38]. *Adverse events* will be defined as the rate of adverse events, such as unplanned extubation and falls, during hospital stay.

### Recruitment timeline

Enrollment and data collection started in November 2021. Recruitment will continue until the target population of 1,452 ICU patients is recruited, which is expected to continue until November 2023.

### Data collection

All assessments will be conducted by research staff with no role in the intervention after undergoing training for conducting research and following standard procedures (S3 File). All data will be collected using standardized forms and entered into a database by two researchers who will conduct extensive error and validity checks. To identify eligible patients, the research staff will review patient medical records and request patients to complete an initial screening assessment. Eligible patients will undergo baseline assessment, which will collect data regarding demographics, medical history, comorbidities, illness severity, extent of delirium using the CAM-ICU, cognitive function using the MMSE, and activities of daily living using the BI. The baseline assessment will be completed within 24 hours of admission to the ICU.

### Data management and monitoring

All data will be collected on printed, pre-coded forms that will be double entered into an electronic database and subjected to extensive error checking and data completeness. The ICUs in the study will have access to only their own data. The supervisors will assume a safety role to monitor the quality and completeness of data at each ICU. They will audit the original data to resolve any problems with data collection and assume the right to terminate the trial for patient safety. All study data will be anonymized and treated confidentially and will not be disclosed to any source other than the research group. For the duration of the study, all paper documents will be locked in cabinets and all electronic data will be entered into a password-protected database.

### Data analysis

All data will be analyzed using SPSS version 26.0 (IBM Corp, Armonk, NY) and R studio version 4.2.0 (Boston, MA, USA). Missing data for important covariates (e.g., age and gender) at random (missing rate < 10%) and outcome variables will be imputed using multiple imputation by chained equations [39, 40]. Statistical analyses will be performed by a statistician blinded to intervention allocations. Continuous variables will be described as means and standard deviations for normally distributed data and as medians and interquartile ranges for non-normally distributed data. Categorical variables will be reported as frequencies and percentages.

Comparisons between groups, including for cognitive load and adherence, will be performed using analysis of variance or the Wilcoxon test, and between-group differences in demographic data will be identified using the Chi-square test. Differences in the incidence of delirium between groups will be analyzed after adjusting for covariables, including age; gender; baseline MMSE score; surgery type; hemoglobin, sodium, and albumin levels; and adherence to delirium interventions, using multi-level logistic regression. The patients will be nested within ICUs for binary endpoints, and the odds ratios (ORs) and 95% confidence intervals (CIs) will be calculated.

Time-to-event results (e.g., mortality) will be described using Kaplan-Meier survival curves, and differences between groups will be compared using log-rank analysis or multi-level Cox regression. The proportional hazards assumption in the Cox model will be evaluated, and the hazard ratios (HRs) and 95% CIs will be calculated. Multiple linear regression will be used for analyzing continuous outcomes, including the duration of ICU delirium and length of ICU and hospital stay. Sensitivity analysis will be performed by assessing whether complete data only and imputation for missing data would yield the same results. All tests will be two-tailed, and a *P*-value < 0.05 will be considered statistically significant.

### Harms and risks

No harm is expected to the patients from participation in the study, and the patient interventions will not pose any additional risks. However, adverse events, such as unplanned extubation, falls, or development of pressure ulcers, can occur even during normal care that can result in patient dysfunction and discomfort. Any adverse events will be recorded and reported to the ethics committee of the hospital as soon as possible.

### Patient and public involvement

Patients and/or the public, including clinical experts, will be involved in the design, implementation, and reporting of this study. A participatory workshop was conducted in September 2021 focused on gathering their opinions and addressing issues in the design of the protocol. Information about the study will be used to inform patients and public representatives, and the results of this study will be published in a peer-reviewed journal.

### Ethical considerations and dissemination

The institutional review board of the two study hospitals approved the study (Approval No. 2021-1-22-5 and No. KS2022029). The trial was registered in the Chinese Clinical Trial Registry (ChiCTR1900023711; registration date June 8, 2019). All methods will be performed in accordance with the Declaration of Helsinki, and the rights of the participants will be ensured by complete adherence to the International Council for Harmonisation of Technical Requirements For Pharmaceuticals for Human Use (ICH) Guideline for Good Clinical Practice.

All eligible patients willing to participate in this study will be required to provide written informed consent, and they will be informed that they can withdraw from the study at any time without reason. All data related to the findings will be shared through the Baidu Network (https://pan.baidu.com/s/1qVeHCHE23n75eGWJBOz1ZQ). All authors will review manuscript drafts, resolve any publication problems, and create all academic posters and papers. All authors propose dissemination of information regarding this study at assisted living facilities and academic conferences.

### Ancillary and post-trial care

As the ICUs in this study will incorporate the intervention protocol into routine care, the patients will not receive additional compensation.

### Validity and reliability/rigor

Before starting the trial, the project director will provide training to the research staff and outcome assessors to minimize errors and improve reliability. All research staff will receive training as required, including provision of theoretical knowledge and use of assessment skills for delirium, to maintain the consistency and reliability of delirium assessment in all included units. The outcome assessors will receive intensive training in the procedures based on standardized patients and in the use of validated and reliable instruments to assess ICU delirium and other outcomes to minimize observer bias.

## Discussion

Delirium causes both short-term and long-term negative health outcomes. The PADIS guideline recommends the use of the ABCDEF bundle, which consists of several nonpharmacologic, multi-component, interprofessional approaches to reduce the incidence of ICU delirium [6]. However, adherence to the bundle interventions remains limited. Therefore, our team developed *AI-AntiDelirium* to reduce the incidence of ICU delirium and improve the adherence of nurses in using the ABCDEF bundle. The use of *AI-AntiDelirium* can timely detect and alert healthcare staff to the possible occurrence of delirium by monitoring real-time data, such as patient vital signs and risk factors, allowing the development of tailored interventions to prevent its occurrence. For patients who are experiencing delirium, *AI-AntiDelirium* can be used to automatically adjust the medication treatment plan and nursing care activities based on patient symptoms and conditions, thereby improving treatment and nursing effectiveness. However, the effectiveness of the use of *AI-AntiDelirium* requires examination. Thus, this study aims to evaluate the effectiveness of the use of *AI-AntiDelirium* in decreasing the incidence of ICU delirium and delirium-related clinical outcomes.

The proposed CRCT has several strengths. First, the trial design is relatively pragmatic and easy to follow. Second, a large sample (1,452 patients) will be recruited to increase the strength of the evidence provided by the results. Third, as the *AI-AntiDelirium* intelligent tool integrates delirium assessment, risk factor assessment, and recommended delirium interventions in one application, it is relatively easy to use. If the trial demonstrates the effectiveness of *AI-AntiDelirium* in reducing the incidence of ICU delirium and promoting adherence to the ABCDEF bundle, it will provide a pragmatic tool (*AI-AntiDelirium*) in delirium management.

Several limitations should be noted. First, this study will be conducted in only two hospitals in a single region, which will limit the generalizability of the results. Second, blinding of patients and healthcare staff is not possible because of the nature of the intervention. To minimize observer bias, validated and reliable instruments will be used to assess ICU delirium and other outcomes, and delirium assessment will be conducted by research investigators independent of the intervention and not involved in patient recruitment. Finally, the study focuses only on the appropriate subset of interventions from the ABCDEF tailored to the patient’s specific risk factors, such as pain, visual impairment, use of anesthetic or sedatives, mechanical ventilation, and infection, and does not include many well-known delirium risk factors, such as noise levels perceived by the patient, use of physical restraint, and use of steroids. In a future study, we will consider the risk factors for ICU delirium more comprehensively.

## Conclusions

Although the ABCDEF bundle has been proven to be effective in reducing the incidence of ICU delirium, the effectiveness of and adherence to the bundle are sub-optimal. If the large-scale *AI-AntiDelirium* trial demonstrates the effectiveness of the *AI-AntiDelirium* tool in reducing the incidence of ICU delirium and promoting adherence to the ABCDEF bundle, it will have a profound impact on the management of ICU delirium in both research and clinical practice.

## Supporting information

S1 File SPIRIT 2013 checklist. (PDF)

S2 File The PADIS guidelines. (PDF)

S3 File Schedule of enrolment, interventions, and assessments. (PDF)
