# Supplementary material for: A cluster-randomized controlled trial of a nurse-led artificial intelligence assisted prevention and management for delirium (AI-AntiDelirium) on delirium in intensive care unit: Study protocol
Source: PLoS One. 2024 Feb 29;19(2):e0298793. doi: 10.1371/journal.pone.0298793 (PMC10903907; doi:10.1371/journal.pone.0298793)
Supplement: S2 File — (PDF) [file pone.0298793.s002.pdf]

S3 file : The Implementation Version of the PADIS Guidelines-For ICU Nurses in Control Group

Part 1 ICU Delirium Assessment Tool

Step 1: Assessment of Awareness Level (RASS)

project	specific description	Score
aggressive behavior	Clearly militant behaviour, violent behaviour, immediate danger to staff	□+4
very restless	Grab or remove drains or various intubations; aggressive	□+3
restless	Frequent aimless movements, resistance to the ventilator	□+2
anxious	Restless, but not violent attacks	□+1
awake and calm	Respond naturally to caregivers	□0
sleepy	Not fully awake, but wakes and maintains arousal by sound stimulation (eyes open/eye contact, >10 seconds)	□-1
mild sedation	Arousal with sound and brief eye contact (<10 seconds)	□-2
Moderate sedation	Movement or eye opening response to sound stimulation (but no eye contact)	□-3
**If RASS ≥ -3, proceed to Step 2: CAM-ICU Assessment**		
deep sedation	No response to sound stimuli, but movement or eye opening after physical stimuli	□-4
Unable to wake up	Does not respond to sound or physical stimuli	□-5
**If RASS score is -4 or -5, stop (patient is unconscious) and reassess later**		
The patient's final RASS score _____ Whether to enter the second step: □Yes □No		

Step 2: Delirium Assessment (CAM-ICU)

**Feature 1: Acute changes or fluctuations in the state of consciousness**		**positive standard**	If positive, tick √ here
▪ Is the patient's state of consciousness different from its baseline state? **OR**<br>▪ Has there been any fluctuation in the patient's state of consciousness in the past 24 hours? Manifested by fluctuations in scores on sedation scales (eg, RASS), GCS, or previous delirium assessments		The answer to any question is "yes" →	
**Feature 2: Attention Disorders**			
**Number-based attention**<br>instruction: Say to the patient, "I'm going to read you 10 numbers. Anytime you hear the number '8,' squeeze my hand." The assessor then reads it in a normal tone of voice The following numbers, each with an interval of 3 seconds.<br>**6 8 5 9 8 3 8 8 4 7**<br>**When the patient does not pinch when the number "8" is read, or when the patient reads other numbers, it is counted as an error.**		Number of errors>2 →	
**Feature 3: Altered level of consciousness**			
Positive if the actual score of the RASS is not awake and calm (0 points)		RASS is not "0" →	
**Feature 4: Confusion in thinking**			
**<u>False Question</u>**<br>1. Can a stone float on water? 2. Are there fish in the sea?<br>3. Is 1 jin heavier than 2 jin? 4. Can you drive nails with a hammer?<br>**Record the number of errors when the patient answers incorrectly**<br><br>**<u>Perform instructions to the</u>**<br>patient to say: "Extend these fingers" (examiner holds out 2 fingers in front of the patient), then say: "Now extend the same number of fingers with the other hand" (this time the examiner does not extend Demonstration)<br>* If the patient has only one hand to move, the second command is changed to ask the patient to "add another finger"<br>**If the patient cannot successfully execute all commands, record 1 error.**		Total number of errors > 1 →	
**CAM-ICU global assessment**<br>**Feature 1 + Feature 2 + Feature 3 or 4 positive = CAM-ICU positive**	Meet the standards →	CAM-ICU positive (delirium present)	
	Not up to standard →	CAM-ICU Negative (no delirium)	

Part 2: ICU Delirium Risk factors assessment

Numbering	entry	Evaluation Rules
A1<br>**Hearing loss**<br>☐	Hearing loss (multiple choice)<br>☐ 1. Normal<br>☐ 2. Mild to moderate<br>☐ 3. Severe	○ Normal (can hear and repeat normal speech at a distance of 1m without difficulty)<br>○ Mild to moderate (there is hearing difficulty in some environments, the speaker needs to increase the volume or slow down the speed of speech so that the patient can hear and repeat the raised voice at a distance of 1m)<br>○ Severe (can't hear the speech at all)<br>**When the hearing loss is severe, it suggests that the patient has a risk factor for delirium "hearing loss" and requires further intervention.**
A2<br>**Vision loss**<br>☐	Vision loss (multiple choice)<br>☐ 1. Normal<br>☐ 2. Mild to moderate<br>☐ 3. Severe	○ Normal (uncorrected visual acuity ≥ 5.0 , tiny details can be seen, including general printed newspapers / books)<br>○ Mild to moderate (naked eye vision is 4.5-4.9 ; objects can be identified; large prints can be read)<br>○ Severe (uncorrected visual acuity ≤ 4.5 ; unable to see objects or difficult to identify objects)<br>**When the vision loss is severe, it suggests that the patient has a risk factor for delirium "visual loss" and requires further intervention.**
A3<br>**Pain**<br>☐	Pain Score: __________ (fill in the blank)	**Assess patient pain according to the Pain Numerical Scale (NRS)**<br>Evaluation criteria: Patients were asked to describe the pain intensity with 0-10, 0 being no pain, 1-3 being mild pain, 4-7 being moderate pain, >7 being severe pain, and 10 being severe pain.<br>**When the pain score is not 0 , it indicates that the patient has the risk factor of delirium "pain" and needs further intervention.**
A4<br>**sedative therapy**<br>☐	**Current medication status (multiple choices)**<br>**A4.1 Benzodiazepine sedative-hypnotics**<br>□ No	When a patient is on either sedative, it suggests that the patient has a risk factor for delirium "sedative therapy" and requires further intervention.

	□ Diazepam<br>□ Midazolam<br>□ lorazepam<br>□ Chlordiazepoxide<br>□ Alprazolam<br>□ Estazolam<br>□ Clonazepam<br>□ Other _____<br><br>**A 4.2 Analgesic and sedative drugs**<br>□ No<br>□ Dexmedetomidine<br>□ Propofol<br>□ Morphine<br>□ Pethidine<br>□ Bunarizine<br>□ Other _____<br><br>**A 4.3 Anesthetics**<br>□ No<br>□ Remifentanil<br>□ Sufentanil<br>□ Midazolam<br>□ Li Yuexi<br>□ Tramadol<br>□ Wan Wen<br>□ Flumazenil<br>□ Naloxone<br>□ Baquting<br>□ A strong dragon<br>□ Ephedrine<br>□ Other _____<br><br>**A 4.4 Anticonvulsants**<br>□ No<br>□ Phenobarbital<br>□ Chloral hydrate<br>□ Magnesium sulfate injection<br>□ Other _____<br><br>**A 4.5 Antipsychotics**<br>□ No<br>□ Chlorpromazine<br>□ Haloperidol<br>□ Olanzapine<br>□ Other _____<br><br>**A 4.6 Anticholinergics**<br>□ No<br>□ Atropine<br>□ Glycopyrrolate<br>□ Penehyclidine<br>□ Scopolamine<br>□ Anisodamine<br>□ Other _____<br><br>**A 4.7 Other psychoactive drugs**	

	______________________________	
**A5** **Invasive ventilator support therapy** ☐	Invasive ventilator support therapy (single choice)<br>☐ 1. Yes<br>☐ 2. No	When the patient was treated with invasive ventilator support, it was suggested that the patient had the risk factor of delirium "invasive ventilator support treatment", and further intervention was required.
A6 Indwelling catheter	Indwelling catheter (single choice)<br>☐ 1. Yes<br>☐ 2. No	When a patient has an indwelling catheter, it is suggested that the patient has a risk factor for delirium "indwelling catheter" and requires further intervention.
A7 **Infection** ☐	A7.1 Infection (single choice)<br>☐ 1. Yes<br>☐ 2. No<br>A7.2 Type of infection (single choice)<br>☐ 1. Respiratory infection<br>☐ 2. Urinary tract infection<br>☐ 3. Wound infection<br>☐ 4. Other _____ (please specify)	When the patient has an axillary temperature ≥ 37.5 and the absorption of heat after surgery is excluded, or the culture of blood, urine and sputum is positive , it indicates that the patient has the risk factor of delirium "infection" and needs further intervention**.**
A8 **Inmobility** ☐	**Does the patient have any of the following:**<br>**(single choice or multiple choice)**<br>☐ 1. None<br>☐ 2. Use constraints<br>☐ 3. Treated with mechanical ventilation<br>☐ 4. Use sedatives<br>☐ 5. Active bleeding<br>☐ 6. Spinal cord injury<br>☐ 7. Open Lumbar Drainage<br>☐ 8. Extracranial Ventricular Drainage<br>☐ 9. Unstable fracture<br>☐ 10. Active myocardial infarction<br>☐ 11. Arrhythmia<br>☐ 12. Respiratory distress	When "none" is selected, it indicates that the patient can perform appropriate early activities ; when any of the other options is selected , it indicates that the patient cannot perform early activities, and there is a risk factor for delirium "inactive" , which requires further intervention.

	☐ 13. Restlessness (RASS score ≥2)<br>☐ 14. Systolic blood pressure <90mmhg or >180mmhg<br>☐ 15. Heart rate <60 beats/min or >130 beats/min<br>☐ 16. Respiratory rate <5/min or >40/min<br>☐ 17. Pulse oximetry <88%<br>☐ 18. Physician ordered bed rest/no activity<br>☐ 19. Use of vasopressors (eg, dopamine, dobutamine, epinephrine, norepinephrine, vasopressin)	
A9<br>**poor sleep quality**<br>☐	**Richard Campbell Sleep Questionnaire Score :**<br>__________	**Richard Campbell Sleep Questionnaire**<br>○ Sleep depth: _____ points (0 points - not good; 100 points - good)<br>○ Easy to fall asleep: _____ points (0 points - not easy; 100 points - easy)<br>○ Easy to wake up during sleep: _____ points (0 points - not easy; 100 points - easy)<br>○ Going back to sleep after waking up: _____ points (0 points - not easy; 100 points - easy)<br>○ Overall quality of sleep: _____ points (0 points - not good; 100 points - good)<br>**When a Richard Campbell Sleep Questionnaire score ≤ 25 indicates the presence of a delirium risk factor of "poor sleep quality", further intervention is required.**
A10<br>**without family accompany**<br>☐	Accompanying family members (single choice)<br>☐ 1. Yes<br>☐ 2. None	When selecting "none" indicates that there is a risk factor for delirium "without family companionship", further intervention is required.

Part 3: Prevention and Management Interventions for Intensive Care Unit Delirium

Risk factors	IF	AND	Nursing Interventions Classification	Nursing Interventions	Frequency
			Usual care	Place the calendar and clock with the correct date and time in the ICU	QD
				Inform the patient of the current time and place in detail	QD
				Turn off the ICU headlights and lower the brightness of corridor lights	qn
				Reduce the alarm sound of medical equipment	qn
				Lower staff's communication voice	qn
				Reschedule medications and procedures to reduce disturbed sleep of patients	qn
Hearing impairment	No hearing aids		Treatment and nursing care	Speak loudly, slowly, and patiently with the patient	QD
			Health education	Advice family members to buy hearing aids for the patient	Visiting hours
	Have hearing aids	at home	Health education	Remind family members to bring hearing aids to the ICU during the next visit	Visiting hours
		in the ICU	Treatment and nursing care	Assist patient to wear hearing aids correctly	QD
			Treatment and nursing care	Assist patient to take hearing aids off correctly	QD
Visual impairment	No eyeglasses		Health education	Advice family members to buy eyeglasses for the patient	Visiting hours
	Have eyeglasses	at home	Health education	Remind family members to bring eyeglasses to the ICU during the next visit	Visiting hours
		in the ICU	Treatment and nursing care	Assist patient to wear eyeglasses correctly	QD
			Treatment and nursing care	Assist the patient to put eyeglasses off correctly	QD
Pain			Treatment and nursing care	Provide non-pharmacological interventions, such as distraction and relaxation therapy	PRN
			Treatment and nursing care	Use analgesics according to the doctor's order	st

Part 3: Prevention and Management Interventions for Intensive Care Unit Delirium (Continued)

Risk factors	IF	AND	Nursing Interventions Classification	Nursing Interventions	Frequency
Use of anesthetic or sedatives			Pharmacotherapy and nursing	Adjust the sedative dose according to RASS and maintain light sedation (RASS score ≥-2)	Q4H & PRN
	Dexmedetomidine was not used	Propofol was not used	Pharmacotherapy and nursing	Remind doctors to use dexmedetomidine or propofol	QD
	Spontaneous awakening trial (SAT) Safety Screen: PASS.		Treatment and nursing care	Implementation of SAT: Reduce the daily dose of sedatives, make fully awake until the patient was able to answer a few simple questions or do some simple command action (i.e. blinking, finger), and then trained doctors or nurses would readjust the dosage of sedative drugs to achieve expected sedation level.	QD
	SAT Safety Screen: Failure.		Monitoring	Observe the patient's respiratory status	PRN
	Have performed SAT	SAT Safety Screen Failure	Pharmacotherapy and nursing	Restart sedation at 50% of the prior dose	QD
Mechanical Ventilation	SBT Safety Screen: PASS.	Doctor's advice: perform SBT	Treatment and nursing care	Conduct SBT according to the doctor's order	QD
		No doctor's advice about performing SBT	Treatment and nursing care	Advise doctors to consider SBTs	st
	SBT Safety Screen: Failure		Monitoring	Observe the patient's respiratory status	PRN
Indwelling catheter			Treatment and nursing care	Remove the catheter as soon as possible	QD
			Treatment and nursing care	Conduct timed urination for the patient	Q4h
Infection			Treatment and nursing care	Reduce invasive operations and avoid unnecessary catheterization	QD
	Respiratory tract infection		Monitoring	Observe the patient's respiratory status	PRN
	Wound infection		Treatment and nursing care	Change dressing for the wound in time to keep the wound clean	QD
	Urinary tract infection		Treatment and nursing care	Advise doctors to remove the catheter as soon as possible	st

Part 3: Prevention and Management Interventions for Intensive Care Unit Delirium (Continued)

Risk factors	IF	AND	Nursing Interventions Classification	Nursing Interventions	Frequency
Immobility	Have activity contraindications		Rest and exercise	Level0: patients are advised to rest in bed and not to be active	st
	No activity contraindications	RASS =-3 or -4 or -5	Rest and exercise	Level1: help patients to do passive range-of-motion exercises, 10 times for each joint	QD
		RASS=-2 or -1 or 0 or +1）	Rest and exercise	Level2: patients are advised to do the active range-of-motion exercises on the bed (10-20 minutes)	QD
			Rest and exercise	Level3: help patients sit on the bedside for 20 minutes	QD
			Rest and exercise	Level4: Assist the patient to stand still at the bedside for 5-10 mins	QD
			Rest and exercise	Level5: Assist patients to walk along the aisle for 5-10 minutes	QD
Sleep disorders（RCSQ ≤25）	-		Rest and exercise	Reduce the time of sleep during the day (< 1/2h)	QD
	Daytime: 8 am-8 pm		Treatment and nursing care	Assist patients to remove earplugs or anti-noise equipment	QD
			Treatment and nursing care	Assist patients to remove the eye mask	QD
	Nighttime: 8 pm-8 am		Treatment and nursing care	Assist patients to wear earplugs or anti-noise equipment	QD
			Treatment and nursing care	Assist patients to wear an eye mask	QD
No family members visit			Social support	Family visits are encouraged	Visiting hours
Delirium（CAM-ICU (+) or ICDSC≥4）			Psychological support	Communicate with patients and comfort them with care to lower their panic and anxiety	PRN
			Social support	Encourage family members to visit patients and help patients with orientation training	Visiting hours
			Health education	Conduct propaganda to the patient's family members, such as symptoms and signs of delirium, treatment nursing interventions	Visiting hours
	No use of non-dexmedetomidine sedatives	RASS=2 or 3 or 4	Pharmacotherapy and nursing	Advise doctors to administer non-benzodiazepine sedatives such as dexmedetomidine to lower the risk of delirium	st
	Invasive MV		Treatment and nursing care	Enforce protective restraint	PRN
