# Supplementary material for: A cluster-randomized controlled trial of a nurse-led artificial intelligence assisted prevention and management for delirium (AI-AntiDelirium) on delirium in intensive care unit: Study protocol
Source: PLoS One. 2024 Feb 29;19(2):e0298793. doi: 10.1371/journal.pone.0298793 (PMC10903907; doi:10.1371/journal.pone.0298793)
Supplement: S3 File — (PDF) [file pone.0298793.s003.pdf]

S4 File. Standard Protocol Items (SPIRIT): details on the scheduled activities related to patient enrollment, interventions, and study variables at different time points.

	Study Period				
	Enrollment	Allocation	Daily Assessment	At discharge of ICU	At discharge of hospital
**Time point**	**-t_1**	**t_0**	**t_1**	**t_2**	**t_3**
**Enrollment**					
Eligibility screen	×				
Informed consent	×				
Allocation		×			
**Interventions**					
The AI-AntiDelirium					
Step1：ICU delirium assessment		×	×		
Step2：Risk factors assessment		×	×		
Step3：Confirmation of nursing care plan		×	×		
Step4：Implementation of nursing activities		×	×		
The PADIS guidelines (Implementation Version)					
Step1：ICU delirium assessment		×	×		
Step2：Risk factors assessment		×	×		
Step3：Implementation of nursing activities		×	×		
**Baseline information**					
**Demographics**		×			
age		×			
gender		×			
body mass index		×			
degree of education		×			
alcohol exposure		×			
smoking exposure		×			
hearing		×			
vision		×			
**Medication history**		×			
**ICU admission diagnosis**		×			
**Comorbidities**		×			
**Current medicine use**		×			
**MMSE**		×			
**CAM-ICU**		×			
**RASS**		×			
**Barthel index**		×			
**APACHE-II**		×			
**NPRS**		×			
**Vital signs**		×			
**Sleep quality**		×			
**Laboratory results**		×			
**Use of sedatives and analgesics**		×			
**Mechanical ventilation**		×			

**Use of physical restraints**	×			
**Indwelling catheter**	×			
**Outcomes**				
Incidence of ICU delirium	×	×	×	
Duration of ICU delirium		×	×	
Length of ICU stay			×	
Length of hospital stay				×
ICU mortality			×	
In-hospital mortality				×
Cognitive function			×	
Activities of daily living			×	
**Adverse events**		×	×	×
**Vital signs**		×	×	×
**Laboratory index**		×	×	×
**Symptoms/Signs**		×	×	×
Difficulty in breathing and shortness of breath		×	×	×
Palpitations, chest pain		×	×	×
Dizziness, syncope, severe headache		×	×	×
Numbness and weakness		×	×	×
Sudden speech difficulties		×	×	×
**Complications (sudden onset during intervention)**		×	×	×
Acute myocardial infarction		×	×	×
Acute cerebral infarction		×	×	×
Acute cerebral hemorrhage		×	×	×
Acute bleeding event		×	×	×
Severe shock from a variety of causes		×	×	×
Acute multiple organ dysfunction syndrome		×	×	×
**Other adverse Events**				
Fall during exercise		×	×	×
Unplanned extubation caused by various reasons		×	×	×
Death		×	×	×
